# Characterization and Biotechnology of Three New Strains of Basidial Fungi as Promising Sources of Biologically Active Substances

**DOI:** 10.3390/biotech14020030

**Published:** 2025-04-25

**Authors:** Maria Alexandrovna Sysoeva, Ilyuza Shamilevna Prozorova, Elena Vladislavovna Sysoeva, Tatyana Vladimirovna Grigoryeva, Ruzilya Kamilevna Ismagilova

**Affiliations:** 1 Food Biotechnology Department, Kazan National Research Technological University, 68 Karl Marx Street, Kazan 420015, Russia; oxygen1130@mail.ru (M.A.S.); inonotus@yandex.ru (E.V.S.); 2 Institute of Fundamental Medicine and Biology, Kazan Federal University, 18 Kremlevskaya Street, Kazan 420008, Russia; tatabio@inbox.ru (T.V.G.); ruz-ismagilova@yandex.ru (R.K.I.)

**Keywords:** basidiomycetes, *Daedaleopsis tricolor*, *Pycnoporellus fulgens*, *Trichaptum abietinum*, solid culture media, cultivation conditions, DNA analysis

## Abstract

The study of new strains of basidiomycetes as sources of biologically active substances is a promising direction in modern biotechnology. This work aims to isolate new cultures of the fungi *Daedaleopsis tricolor*, *Pycnoporellus fulgens* and *Trichaptum abietinum* from natural fruiting bodies and to improve their growth conditions on solid nutrient media. The identification of fungi was performed based on their morphological features and using the Sanger sequencing method. Cultivation was carried out by placing inoculum in the middle of a Petri dish and at the edge, which provided a more comprehensive definition of the characteristics of colonies and fungus hyphae. New strains were registered in Genbank Overview. The optimal cultivation temperature was 27 °C for all studied strains. The highest radial growth was observed on synthetic medium for *D. tricolor* (5.26 mm/day) and *T. abietinum* (7.5 mm/day)*,* and on synthetic medium with lignin for *P. fulgens* (2.98 mm/day). The biomass amount of *D. tricolor* KS11 was 133.25 mg at 9 days of cultivation, that of *P. fulgens* KS12 was 86.73 mg at 16 days, and that of *T. abietinum* KS10 was 227.33 mg at 6 days. New strains of fungi can be used to obtain biologically active substances for the food, pharmaceutical and cosmetic industries.

## 1. Introduction

Basidiomycetes cultivation in artificial conditions to studying the substances they synthesize is an urgent task, since their fruiting bodies, growing in nature, contain biologically active components that are promising for the production of supplements and medicines [1,2,3,4,5,6,7,8,9,10,11,12]. Currently, the most well-known cultivated mushrooms are *Ganoderma lucidum* (reishi), *Lentinus edodes* (shiitake), *Cordyceps militaris* (military cordyceps), *Inonotus obliquus* (chaga) and *Trametes versicolor* (turkey tail). They contain biologically active metabolites such as polysaccharides, proteins, pigments, phenolic compounds and terpenoids [13,14,15,16,17,18,19,20,21,22,23], and are used in the pharmaceutical, food and cosmetic industries [17,24,25,26,27,28,29]. The expansion of the spectrum of higher fungi that are efficient producers of active components is of practical interest. For example, fungi such as *Trichaptum abietinum*, *Daedaleopsis tricolor* and *Pycnoporellus fulgens* (*Hapalopilus fibrillosus*) are widespread in Europe and Asia and in some other regions with a temperate climate worldwide [30,31,32,33,34,35,36,37,38]. At the same time, there are few data on these basidiomycete members in the literature, and there are only a few publications about their cultivation [33,39,40,41,42,43].

It is known that the mycelium of *D. tricolor* contains proteins, polysaccharides, phenolic compounds, flavonoids, terpenoids and carotenoids. Aqueous extracts from the mycelia of *D. tricolor* exhibit antitumor activity against Hep-2 cells (IC_50_—100–115 μg/mL) and have high antioxidant and antifungal activity [42,43]. *P. fulgens* is interesting for biotechnology because it produces lignin-modifying and cellulolytic enzymes, including active lignin peroxidases and H_2_O_2_-producing oxidases [40]. *T. abietinum* is known as a producer of lignin-modifying oxidoreductases, such as laccase and manganese peroxidase [33,41]. Basidiomycetes *D. tricolor*, *P. fulgens* and *T. abietinum* produce various biologically active compounds. This demonstrates their biotechnological potential and their potential for use in the development of biologically active additives and medicines based on them.

The isolation and study of new strains will allow us to search for and select fast-growing basidiomycetes as producers for use in industrial biotechnology.

The aim of this work is to introduce new strains of *Daedaleopsis tricolor*, *Pycnoporellus fulgens* and *Trichaptum abietinum* into culture and to identify their culture conditions on solid nutrient media, which ensure intensive growth and biomass accumulation.

## 2. Materials and Methods

### 2.1. Isolation of Pure Culture

*Trichaptum abietinum* fungi were collected from spruce (*Picea abies* (L.) H. Karst) in a mixed forest in the Mari El Republic near Volzhsk (56°04′04″ N and 48°20′11″ E) in September 2019. *Daedaleopsis tricolor* and *Pycnoporellus fulgens* were collected from birch trees (*Betula* L., 1753) in the Republic of Tatarstan near Kazan (55°57′17″ N and 49°09′04″ E) in July 2021. These forests contain coniferous trees (pine, spruce), deciduous trees (birch, maple, oak, linden) and various shrubs. In the Republic of Mari El near Volzhsk, in the year 2019, the weather conditions were as follows. In July, the average temperature was 18.56 °C, with a total of 151.7 mm of precipitation and an average humidity of 80.9%. In August, the average temperature was 15.81 °C, with a total of 69.06 mm of precipitation and an average humidity of 81.3%. In September, the average temperature was 11.11 °C, with a total of 41.65 mm of precipitation and an average humidity of 80.6% [44]. In 2021, the weather conditions in the Republic of Tatarstan near Kazan were as follows. The average temperature in May was 18.1 °C, with 20 mm of precipitation and 60% humidity. In June, the average temperature was 22.4 °C, with 21 mm of precipitation and 57% humidity. July saw an average temperature of 22 °C, 81 mm of precipitation and 63% humidity [45,46].

Pure fungal cultures were isolated using the standard tissue method [33,47]. The inoculum was cultivated on the surface of a dense potato glucose medium at 27 ± 2 °C for 5–7 days in a thermostat (TS-1/80 SPU, Smolensk, Russia). The isolated pure cultures were stored in a test tube with slant agar at 4 ± 2 °C [47].

### 2.2. Identification of Fungal Strains

The strains were identified based on their micro- and macromorphological features. Microscopy of the mycelium was performed as in [33] at 400 magnification using a microscope (MC 100 (LCD PC), Vienna, Austria).

The mycelium was inoculated at the center or at the edge of a Petri dish to determine the time of colonization of the Petri dish and to identify the morphological features of the fungal culture [33].

Primary nucleotide sequences of DNA were determined using Sanger sequencing. The sequencing reaction was performed with gene-specific primers ITS1 (5-TCCGTAGGTGAACCTGCGG-3) and ITS4 (5-TCCTCCGCTTATTGATATGC-3) and a Big Dye Terminator v 3.1 Cycle Sequencing Kit (Applied Biosystems^TM^, Foster City, CA, USA). A total of 2–5 µL of purified product of polymerase chain reaction (PCR) in an amount of 10 to 50 ng (depending on the length of the PCR product) was mixed with 1 µL of 3.2 pmol primer, 0.8 µL of Ready Reaction Mix, 1.6 µL of 5X Sequencing Buffer and water to a volume of 10 µL. Reading was carried out with both primers (forward and reverse). The reaction was carried out using a Veriti amplifier (Applied Biosystems^TM^, Foster City, CA, USA) according to the following temperature protocol: preliminary denaturation at 96 °C for 1 min, 26 cycles at 96 °C for 10 s, 50 °C for 5 s, and 60 °C for 4 min. The sequencing of PCR products was performed on an ABI PRISM 3730 automatic sequencer (Applied Biosystems^TM^, Foster City, CA, USA) at the Interdisciplinary Center for Collective Use of Kazan Federal University [47].

### 2.3. Selection of Nutrient Media and Temperature for Cultivation

The fungal strains were cultivated on the following agar nutrient media: (1) Sabouraud agar (State Research Center for Applied Biotechnology and Microbiology, Obolensk, Russia); (2) Czapek agar (BioCompass-S, Moscow, Russia); (3) potato glucose agar (potato—200 g/L; glucose—20 g/L; microbiological agar—20 g/L); (4) glucose peptone agar (glucose—30 g/L; peptone—5 g/L; potassium dihydrogen phosphate—0.5 g/L; magnesium sulfate—0.5 g/L; microbiological agar—20 g/L); (5) synthetic medium (glucose—20 g/L; yeast extract—5 g/L; potassium dihydrogen phosphate—0.5 g/L; magnesium sulfate—0.5 g/L; microbiological agar—20 g/L); (6) synthetic medium with lignin (glucose—20 g/L; yeast extract—5 g/L; lignin (Entegnin (hydrolytic lignin), “GNTS PM Farma”, Moscow, Russia)—0.01 g/L; potassium dihydrogen phosphate—0.5 g/L; magnesium sulfate—0.5 g/L; microbiological agar—20 g/L). The diameter of the inoculation block was up to 5 mm. Fungus cultivation was carried out in a thermostat (TS-1/80 SPU, Smolensk, Russia) at 27 ± 2 °C for 6–17 days.

To select the optimal growth temperature for the fungi, they were cultivated at 15, 24, 27 and 37 °C for 6 days on a synthetic nutrient medium (*D. tricolor* KS11 and *T. abietinum* KS10) and a synthetic nutrient medium with lignin (*P. fulgens* KS12).

### 2.4. Determination of Radial Growth and Biomass Yield

To determine the radial growth of the fungal colony, the inoculum was placed in the middle of the Petri dish with synthetic nutrient medium (*D. tricolor* KS11 and *T. abietinum* KS10) and a synthetic nutrient medium with lignin (*P. fulgens* KS12). Two mutually perpendicular diameters of colonies were measured after 6 days of cultivation for further calculation of their radius and radial growth as described in [48]. The mycelium grown as described in Section 2.3 was separated from the solid nutrient medium by melting it in a water bath, and then washed with water. The separated, washed fungal mycelium was dried at 40 ± 2 °C by the convective–conductive method in an electric dryer (Oberhof Fruchttrockner B-53, Foshan, China). The amount of biomass was determined by the gravimetric method [47].

### 2.5. Statistical Analysis

Each experiment and measurement was repeated three times, and the results are expressed as the average value ± standard deviation (*p* < 0.05). The statistical analysis was conducted with Statistica 13 with a data analysis toolkit.

## 3. Results and Discussion

### 3.1. Isolation and Identification of Fungal Strains of Daedaleopsis tricolor, Pycnoporellus fulgens and Trichaptum abietinum

**Figure 1:**
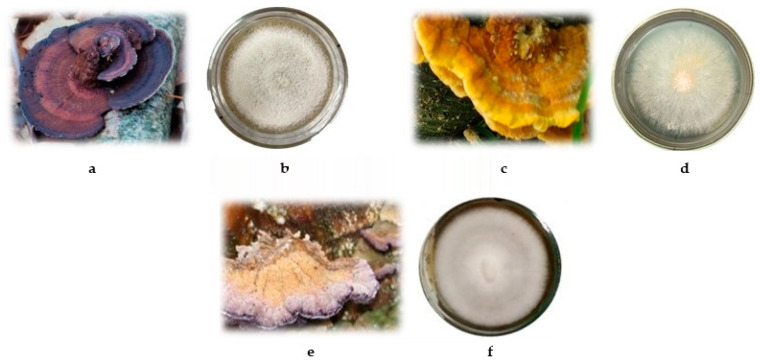
Fruiting bodies and aerial mycelium of fungi: (**a**,**b**) *Daedaleopsis tricolor*; (**c**,**d**) *Pycnoporellus fulgens*; (**e**,**f**) *Trichaptum abietinum*.

The isolated pure cultures were studied for macro- and micromorphology. The natural fruiting bodies of fungi and their colonies grown on the surface of a dense potato-glucose medium are shown in Figure 1.

*D. tricolor* forms felt colonies. The mycelium is loose and white, with compacted areas with beige-brown pigmentation appearing with age. There is a zone around the inoculum that has the lowest mycelial density, with a diameter of 5 mm. The edge of the colony is smooth and rounded (Figure 1b). The mycelium of *P. fulgens* is cotton-like, uneven in density, and fluffy. The edge of the colony is even. The colony is white, but in the center, there is a light yellow pigment with an orange reverse (Figure 1d). The mycelium of *T. abietinum* is white, with a yellow tint. The strain forms concentric ring-like aerial colonies with smooth edges (Figure 1f). All isolated cultures have a pleasant mushroom smell.

**Figure 2:**
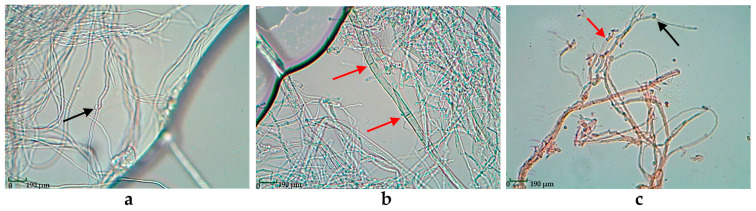
Microscope photographs of mycelium at 400× magnification: (**a**) *Daedaleopsis tricolor*; (**b**) *Pycnoporellus fulgens*; (**c**) *Trichaptum abietinum* (the red arrows show the septa, and the black arrows show the buckles).

According to microscopic studies (Figure 2a), the hyphae of *D. tricolor* are branched and thin-walled with buckles. The aerial mycelium of *P. fulgens* is formed by septate thick- and thin-walled hyphae without buckles (Figure 2b). The hyphae of *T. abietinum* are septate and thick- or thin-walled with buckles (Figure 2c).

The morphological characteristics of the isolated strains are generally consistent with the information available in the scientific literature [33,39]. Scientists from Italy and Spain characterized colonies of *T. abietinum* grown on agarized malt extract (2%) at 25 °C as follows. The mycelium is aerial, cottony and flocculent with a white, pale or transparent color. The color of the reverse side of the Petri dish is the same. The hyphae are thin-walled and septate, with clamps and numerous short branches [33]. Other authors [49] describe the mycelium of *T. abietinum* as uncolored, with the colony exhibiting pressed edges, while *D. tricolor* is described as having aerial mycelium with pressed edges, ranging in color from white to brown [49]. Variations in the morphological features of the cultures may be due to the locations of the collection of natural fruiting bodies, as well as differences in the climate and cultivation conditions.

To confirm the morphological identification of the fungi, sequencing using the Sanger method was performed.

**Figure 3:**
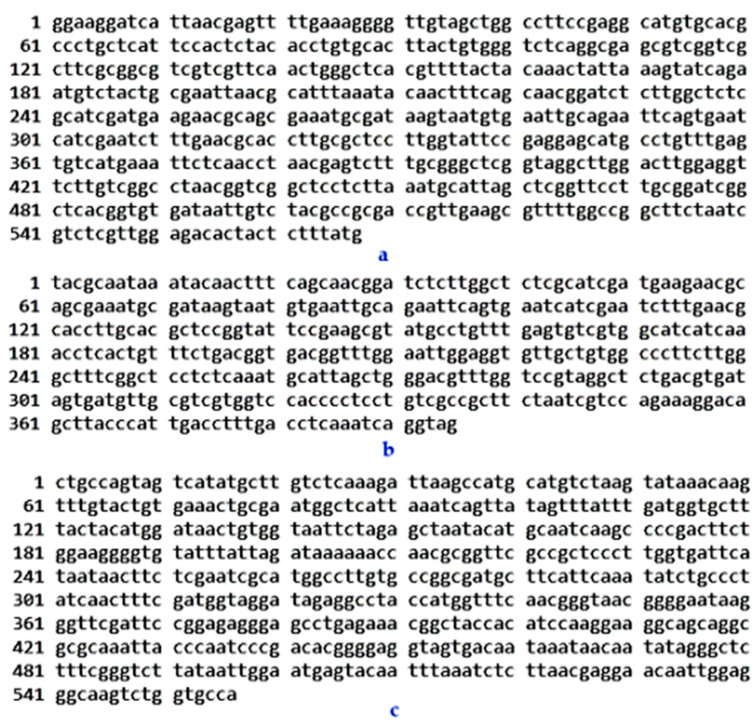
Oligonucleotide sequences: (**a**) *Daedaleopsis tricolor*; (**b**) *Pycnoporellus fulgens*; (**c**) *Trichaptum abietinum*.

A high-quality sequence of 567 oligonucleotide sequences was obtained for *D. tricolor*, of 395 for *P. fulgens*, and of 556 for *T. abietinum* (Figure 3).

Using the NCBI database, a sequence similarity of higher than 99% was established for the isolated cultures with the sequences of the strains *D. tricolor*, *P. fulgens* and *T. abietinum*.

The cultures were deposited in the Genbank Overview database. The geographical information, assigned numbers and fungal strains are presented in Table 1.

As a result, it was established that the isolated strains KS11, KS12 and KS10 actually belong to *D. tricolor*, *P. fulgens* and *T. abietinum*, respectively. The new strains were registered in the Genbank Overview database with the numbers OR804093 (*Daedaleopsis tricolor* KS11), OR805526 (*Pycnoporellus fulgens* KS12) and OR610852 (*Trichaptum abietinum* KS10).

### 3.2. Selection of Nutritional Media for Culturing Strains of Daedaleopsis tricolor KS11, Pycnoporellus fulgens KS12 and Trichaptum abietinum KS10

The development of one universal medium suitable for even related species is impossible, because the morphogenetic characteristics of different strains vary. Therefore, the selection of a nutrient medium for each new strain is a necessary step. To achieve this aim, nutrient media frequently used for basidiomycetes cultivation were employed [41,47,50,51,52,53,54]. The control nutrient medium for fungus cultivation was potato glucose agar, since new strains are isolated on it and museum cultures are stored on it. The other nutrient media used differ in their composition and the concentration of nitrogen and carbon-based substances.

Sabouraud agar contains the highest concentration of glucose and several nitrogen sources [54]. Cultivation on this nutrient medium can show how the culture will grow under conditions of excess nutrient components. Czapek agar includes another carbon-containing substrate—sucrose and an inorganic form of nitrogen [50]. The source of nitrogen in the glucose peptone agar is peptone. The synthetic media contained glucose as a carbon source and yeast extract as a source of nitrogen and B vitamins. The synthetic media differ from each other in that the second synthetic medium contained a source of lignin. The selected composition of nutrient media will help us to establish the optimal source of carbon and nitrogen for each fungal culture.

**Figure 4:**
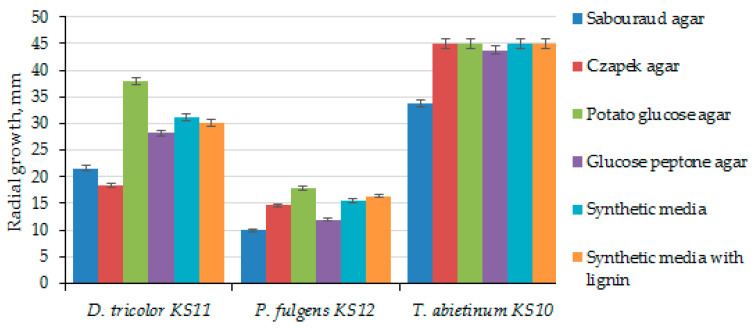
Radial growth of fungi on 6th day of cultivation.

The growth of the studied cultures was assessed based on radial growth and biomass accumulation (Table 2). Figure 4 shows the radiuses of 6-day colonies of *D. tricolor* KS11, *P. fulgens* KS12 and *T. abietinum* KS10, obtained on solid nutrient media.

The amount of mycelium accumulated by the studied basidiomycetes during their cultivation on different solid nutrient media was also determined. The results are presented in Table 2.

All the studied basidiomycetes grew well on potato glucose medium. The highest radial growth by the sixth day of cultivation (Figure 4) was observed for *T. abietinum* KS10 on almost all solid media, with the exception of glucose peptone agar and Sabouraud agar. It can be assumed that the slow growth of the culture on Sabouraud agar is associated with its high glucose content, as well as with excess nitrogen sources [54]. The optimal growth rate for *T. abietinum* KS 10 was observed on Czapek agar, potato glucose agar, synthetic medium and synthetic medium with lignin and was 7.5 mm/day. Other strains of this species grown on a medium selected for them (malt extract agar) had a lower growth rate. For example, *T. abietinium* 0110 grew at 5.8 mm/day and *T. abietinium* 5406 at almost 2 mm/day.

Intensive growth (6.32 mm/day) of *D. tricolor* KS11 was observed on potato glucose agar (Figure 4). Similar data (7 ± 2 mm/day) regarding the growth rate of this fungus on standard malt extract agar medium were presented in the work [39]. The slow growth of *D. tricolor* KS11 on Czapek agar with sucrose as a carbon source indicates that this strain may not metabolize sucrose well. This is probably due to the inactivity or absence of β-glycosidase in this fungus [41].

The maximum radial growth (17.88 ± 0.45 mm) of the colony of *P. fulgens* KS12 was achieved on the potato glucose agar (Figure 4), and the minimum (9.88 ± 0.2 mm) on the Sabouraud agar. As was mentioned earlier, growth inhibition on this solid nutrient medium occurs due to excess carbon- and nitrogen-containing substrates [53,54]. The results obtained are consistent with the literature data [40], where the growth rate of *P. fulgens* on the malt extract agar was average (complete overgrowth of the Petri dish in 2–3 weeks or more).

The cultivation of *D. tricolor* KS11 and *T. abietinum* KS10 on Sabouraud agar allowed us to obtain the highest amount of biomass (168.67 ± 9.87 mg for 13 days and 262.33 ± 52.54 mg for 9 days, respectively), but over a longer period compared to cultivation on other media. Intensive accumulation of the aerial mycelium of these basidiomycetes was also observed on synthetic medium, while the biomass amount of *T. abietinum* KS10 was 1.4 times greater and its cultivation duration was 3 days faster (171.50 ± 60.10 for 6 days) compared to *D. tricolor* KS11 (125.00 ± 24.58 mg for 9 days).

According to the literature data [47], the addition of lignin sources to the nutrient medium of basidiomycetes can increase their growth rate and accumulation of biomass. Although it is known that *P. fulgens* [40] and *T. abietinum* [33,41] synthesize and use lignin-modifying and cellulolytic enzymes in their metabolism, as demonstrated in Table 2, only *P. fulgens* KS12 requires lignin. This fungus on the synthetic medium with lignin accumulated a higher amount of biomass (81.33 ± 16.01 mg) compared to its growth on other media.

The fungal cultures under investigation can be ranked based on their radial growth and biomass production, with *Trichaptum abietinum* KS10 leading the way, followed by *Daedaleopsis tricolor* KS11 and *Pycnoporellus fulgens* KS12.

Based on the obtained results, the following nutrient media were selected for the cultivation of basidiomycetes: for *D. tricolor* KS11 and *T. abietinum* KS10, the synthetic medium, and for *P. fulgens* KS12, the synthetic medium with lignin. In these fungi, rapid growth and biomass accumulation were observed on these nutrient media.

### 3.3. Effect of Temperature on Growth of Daedaleopsis tricolor KS11, Pycnoporellus fulgens KS12 and Trichaptum abietinum KS10

**Figure 5:**
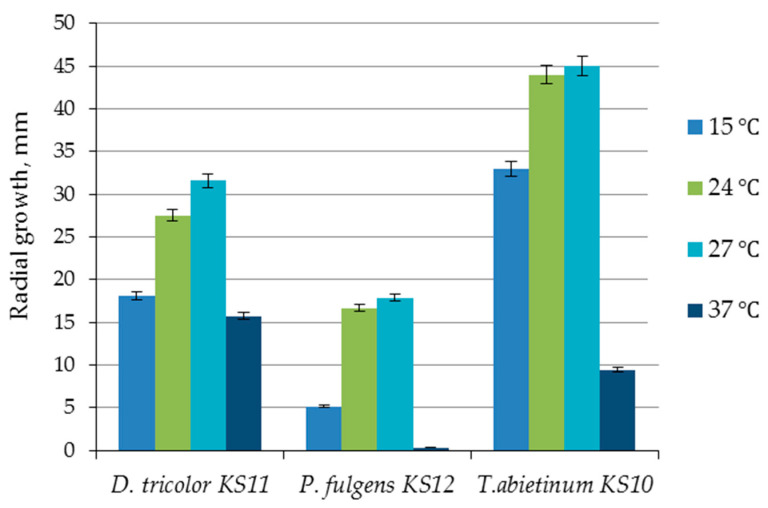
Radial growth of fungi on the 6th day of cultivation on synthetic medium (*D. tricolor* KS11 and *T. abietinum* KS10) and on synthetic medium with lignin (*P. fulgens* KS12) under different temperature conditions.

The selection of the optimal temperature for the cultivation of new fungal strains was carried out on the synthetic medium (*D. tricolor* KS11 and *T. abietinum* KS10) and on the synthetic medium with lignin (*P. fulgens* KS12) at 15 °C, 24 °C, 27 °C and 37 °C. The radial growth of the colonies on the sixth day of cultivation is shown in Figure 5.

The highest radial growth rates of 5.26 mm/day (*D. tricolor* KS11), 2.98 mm/day (*P. fulgens*) and 7.5 mm/day (*T. abietinum* KS10) were observed at 27 °C.

The results of determining the amount of biomass during the cultivation of *D. tricolor* KS11, *P. fulgens* KS12 and *T. abietinum* KS10 at various temperatures are presented in Table 3.

The optimal growth temperature, selected based on the radial growth value (Figure 5) and the amount of accumulated biomass (Table 3) for all studied strains, was 27 °C, which is consistent with the literature data. The growth of all studied basidiomycetes usually occurred at about 25 ± 2 °C [39,40,41,42,43].

Thus, at the optimal cultivation temperature, on selected nutrient media, for each fungus, the amount of biomass of *D. tricolor* KS11 was 133.25 mg after 9 days, of *P. fulgens* KS12 was 86.73 mg after 16 days and of *T. abietinum* KS10 was 227.33 mg after only 6 days.

### 3.4. A Detailed Description of the Morphological Features of the Colonies of Daedaleopsis tricolor KS11, Pycnoporellus fulgens KS12 and Trichaptum abietinum KS10

In order to study the morphological features of the isolated strains in more detail, they were cultivated by seeding the inoculum at the edge of a Petri dish under selected optimal conditions (temperature, nutrient medium). This cultivation approach made it possible to comprehensively describe in more detail the morphological features of the colonies, as well as their characteristics and growth.

**Figure 6:**
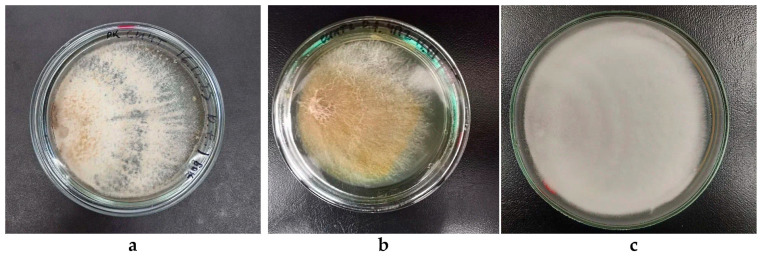
Colonies of the fungus strains inoculated at the edge of a Petri dish at the end of cultivation: (**a**) *Daedaleopsis tricolor* KS11 (11 days); (**b**) *Pycnoporellus fulgens* KS12 (17 days); (**c**) *Trichaptum abietinum* KS10 (9 days).

The cultivation process was conducted under the following conditions: A temperature of 27 °C was maintained for all fungal species. *D. tricolor* KS11 and *T. abietinum* KS10 were cultivated on a synthetic medium, while *P. fulgens* KS12 was grown on a medium supplemented with lignin. The duration of cultivation of *D. tricolor* KS11 with this inoculum arrangement was 11 days, that of *P. fulgens* KS12 was 17 days, and that of *T. abietinum* KS10 was 9 days. Colonies of *D. tricolor* KS11, *P. fulgens* KS12 and *T. abietinum* KS10 are shown in Figure 6.

*D. tricolor* KS11 forms a velvety colony type. The mycelium is dense, with long tangled hyphae. The colony is white during the first 4 days of cultivation, and on the fifth day, the colony acquires a brown-gray pigment, while the edge of the colony remains white. The edge of the colony is uniform. It has a distinct mushroom odor (Figure 6a).

The aerial mycelium of *P. fulgens* KS12 forms a loose, cobweb-like film with long tangled hyphae. The colony colors are yellow, ochre, orange and white in places. The edge of the colony is uneven, and the pigment is irregularly distributed (Figure 6b).

*T. abietinum* KS10 forms aerial, felt-like colonies with a white color. The hyphae are short, low. The edge is smooth. Concentric zonation is observed (Figure 6c). It has a pronounced mushroom smell.

The colony of *D. tricolor* KS11 grown in this way had more pronounced zonality and ray-shaped growth of its hyphae, as well as pigment allocation, compared to the standard seeding of the inoculum in the center of the dish. The mycelial growth of *P. fulgens* KS12 and *T. abietinum* KS10 was not significantly different between two inoculation methods. When inoculated at the edge of a Petri dish *T. abietinum* KS10 showed the shortest time for overgrowing the surface of the nutrient medium, which confirms its rapid growth rate.

The growth rate and ability to accumulate biomass on inexpensive nutrient media are key indicators that determine the possibility of developing profitable industrial technologies for producing biologically active substances and supplements using fungi [53]. We plan to further investigate the biologically active compounds synthesized by these new strains and determine whether valuable components should be isolated or whether their biomass can be used as supplements.

## 4. Conclusions

New strains of basidiomycetes *Daedaleopsis tricolor* KS11, *Pycnoporellus fulgens* KS12 and *Trichaptum abietinum* KS10 were introduced into culture and deposited in the Genbank database. The initial identification of pure fungus cultures was based on an analysis of macro- and micromorphological features, followed by DNA sequencing for confirmation. A solid culture medium and temperature were selected to meet the nutrient requirements of newly isolated strains and promote their rapid growth and biomass accumulation. It was determined that the suitable solid nutrient medium for the growth of *D. tricolor* KS11 and *T. abietinum* KS10 was the synthetic medium, while for *P. fulgens* KS12, it was the synthetic medium with lignin. All basidiomycete cultures should be cultivated at 27 °C.

The fungus *T. abietinum* KS10 is a productive and fast-growing strain, since its cultivation duration on a synthetic solid nutrient medium was 6 days. This was 3 and 10 days less compared to the cultivation time of *D. tricolor* KS11 on the same medium and *P. fulgens* KS12 on the synthetic medium with lignin. The amount of accumulated mycelium of *T. abietinum* KS10 by the end of cultivation (227.3 mg) was 1.7 and 2.6 times higher compared to that of *D. tricolor* KS11 and *P. fulgens* KS12.

## Figures and Tables

**Table 1:** Geographic data and assigned GenBank numbers of newly isolated fungal strains.

Fungus	Strain	Year of Collection	Place of Collection	Year of Deposition	GenBank Number
*Daedaleopsis tricolor*	KS11	2021	Republic of Tatarstan	2023	OR804093
*Pycnoporellus fulgens*	KS12	2021	Republic of Tatarstan	2023	OR805526
*Trichaptum abietinum*	KS10	2019	Republic of Mari El	2023	OR610852

**Table 2:** Mycelial counts of *D. tricolor* KS11, *P. fulgens* KS12 and *T. Abietinum* KS10 on different solid nutrient media at 27 °C.

Nutrient Media	*D. tricolor* KS11			*P. fulgens* KS12			*T. abietinum* KS10		
	Days	Amount of Biomass		Days	Amount of Biomass		Days	Amount of Biomass	
		mg	mg/cm^2^		mg	mg/cm^2^		mg	mg/cm^2^
Sabouraud agar	13	168.67 ± 9.87	2.76 ± 0.18	-	-	-	9	262.33 ± 52.54	4.13 ± 0.83
Czapek agar	16	33.00 ± 18.03	0.54 ± 0.28	17	2.00 ± 1.00	0.04 ± 0.02	6	1.00 ± 0.05	0.02 ± 0.01
Potato glucose agar	9	85.00 ± 9.17	1.34 ± 0.14	12	30.00 ± 0.75	0.47 ± 0.02	6	127.67 ± 23.35	2.01 ± 0.37
Glucose peptone agar	9	57.00 ± 3.61	0.96 ± 0.10	17	79.67 ± 22.85	1.33 ± 0.33	7	108.00 ± 44.51	1.70 ± 0.70
Synthetic medium	9	125.00 ± 24.58	1.97 ± 0.39	16	70.00 ± 10.00	1.01 ± 0.01	6	171.50 ± 60.10	2.70 ± 0.95
Synthetic medium with lignin	9	125.33 ± 24.95	2.04 ± 0.28	16	81.33 ± 16.01	1.37 ± 0.25	6	157.00 ± 26.21	2.47 ± 0.41

**Table 3:** Mycelial counts of *D. tricolor* KS11, *P. fulgens* KS12 and *T. abietinum* KS10 under different temperature conditions.

Temperature, °C	*D. tricolor* KS11			*P. fulgens* KS12			*T. abietinum* KS10		
	Days	Amount of Biomass		Days	Amount of Biomass		Days	Amount of Biomass	
		mg	mg/cm^2^		mg	mg/cm^2^		mg	mg/cm^2^
15	10	84.75 ± 21.90	1.33 ± 0.34	-	-	-	9	195.33 ± 2.08	3.07 ± 0.03
24	10	130.25 ± 25.12	2.05 ± 0.40	13	70.00 ± 17.32	1.23 ± 0.31	7	189.67 ± 20.21	3.13 ± 0.32
27	9	133.25 ± 8.66	2.10 ± 0.14	16	86.73 ± 11.01	1.42 ± 0.24	6	227.33 ± 36.53	3.58 ± 0.57
37	Inhibits growth								

## Data Availability

The original contributions presented in this study are included in the article. Further inquiries can be directed to the corresponding author.
